# Supplementary material for: Causal Relationship between Aging and Anorexia Nervosa: A White-Matter-Microstructure-Mediated Mendelian Randomization Analysis
Source: Biomedicines. 2024 Aug 16;12(8):1874. doi: 10.3390/biomedicines12081874 (PMC11351342; doi:10.3390/biomedicines12081874)
Supplement: Supplementary file 1 [file biomedicines-12-01874-s001.zip › Supplementary Figure/Figure S4/Singe SNP plot of MR analysis for TL to ubm-b-1638.pdf]

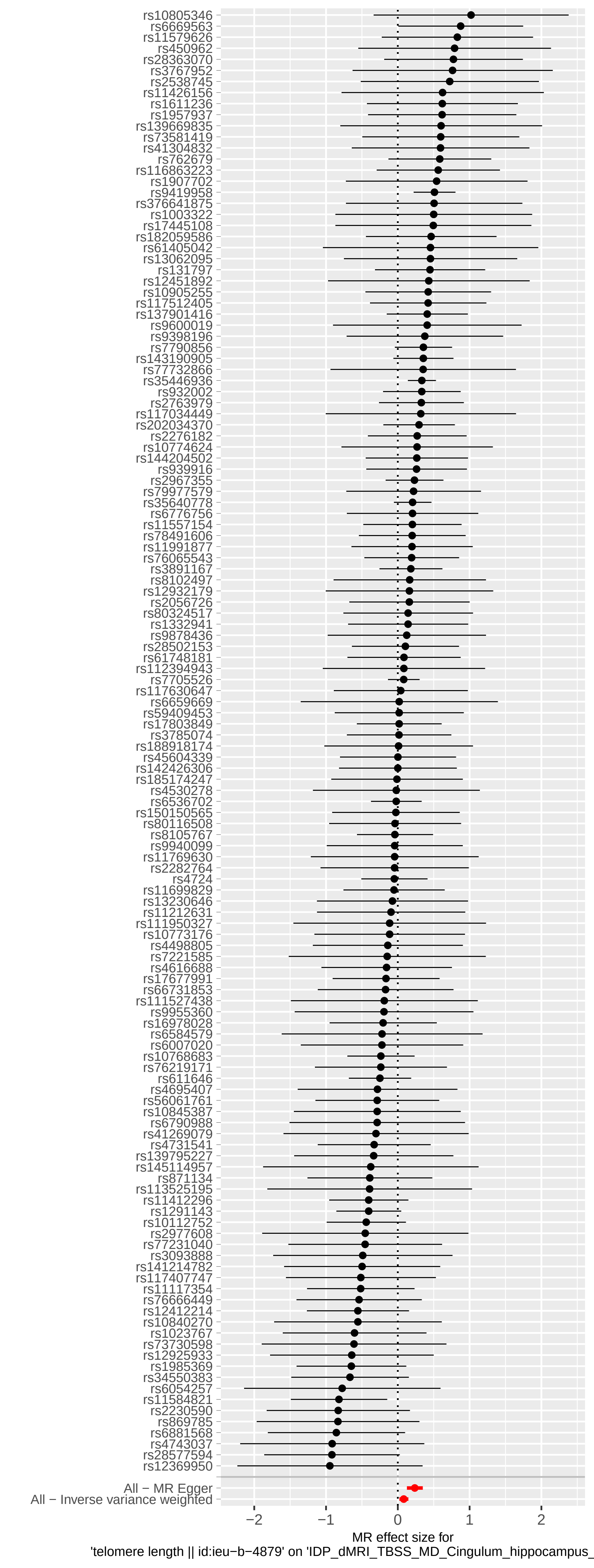

rs10805346
rs6669563
rs11579626
rs450962
rs28363070
rs3767952
rs2538745
rs11426156
rs1611236
rs1957937
rs139669835
rs73581419
rs41304832
rs762679
rs116863223
rs1907702
rs9419958
rs376641875
rs1003322
rs17445108
rs182059586
rs61405042
rs13062095
rs131797
rs12451892
rs10905255
rs117512405
rs137901416
rs9600019
rs9398196
rs7790856
rs143190905
rs77732866
rs35446936
rs932002
rs2763979
rs117034449
rs202034370
rs2276182
rs10774624
rs144204502
rs939916
rs2967355
rs79977579
rs35640778
rs6776756
rs11557154
rs78491606
rs11991877
rs76065543
rs3891167
rs8102497
rs12932179
rs2056726
rs80324517
rs1332941
rs9878436
rs28502153
rs61748181
rs112394943
rs7705526
rs117630647
rs6659669
rs59409453
rs17803849
rs3785074
rs188918174
rs45604339
rs142426306
rs185174247
rs4530278
rs6536702
rs150150565
rs80116508
rs8105767
rs9940099
rs11769630
rs2282764
rs4724
rs11699829
rs13230646
rs11212631
rs111950327
rs10773176
rs4498805
rs7221585
rs4616688
rs17677991
rs66731853
rs111527438
rs9955360
rs16978028
rs6584579
rs6007020
rs10768683
rs76219171
rs611646
rs4695407
rs56061761
rs10845387
rs6790988
rs41269079
rs4731541
rs139795227
rs145114957
rs871134
rs113525195
rs11412296
rs1291143
rs10112752
rs2977608
rs77231040
rs3093888
rs141214782
rs117407747
rs11117354
rs76666449
rs12412214
rs10840270
rs1023767
rs73730598
rs12925933
rs1985369
rs34550383
rs6054257
rs11584821
rs2230590
rs869785
rs6881568
rs4743037
rs28577594
rs12369950
All − MR Egger
All − Inverse variance weighted
−2
−1
0
1
2
MR effect size for
'telomere length || id:ieu-b-4879' on 'IDP_dMRI_TBSS_MD_Cingulum_hippocampus_
